# A Bromodomain-Containing Protein 4 (BRD4) Inhibitor Suppresses Angiogenesis by Regulating AP-1 Expression

**DOI:** 10.3389/fphar.2020.01043

**Published:** 2020-07-10

**Authors:** Zijun Zhou, Xiaoming Li, Zhiqing Liu, Lixun Huang, Yuying Yao, Liuyou Li, Jian Chen, Rongxin Zhang, Jia Zhou, Lijing Wang, Qian-Qian Zhang

**Affiliations:** ^1^ School of Life Sciences and Biopharmaceutics, Guangdong Pharmaceutical University, Guangzhou, China; ^2^ Guangdong Province Key Laboratory for Biotechnology Drug Candidates, Guangdong Pharmaceutical University, Guangzhou, China; ^3^ Chemical Biology Program, Department of Pharmacology and Toxicology, University of Texas Medical Branch, Galveston, TX, United States; ^4^ Department of Pathology, People’s Hospital of Baoan District, Affiliated Baoan Hospital of Shenzhen, Southern Medical University, The Second Affiliated Hospital of Shenzhen University, Shenzhen, China

**Keywords:** bromodomain-containing protein 4 (BRD4), BRD4 inhibitors, BD domains, angiogenesis, AP-1 transcription factors

## Abstract

Angiogenesis dysregulation contributes to inflammation, infections, immune disorders, and carcinogenesis. Bromodomain-containing protein 4 (BRD4) is an epigenetic reader that recognizes histone proteins and acts as a transcriptional regulator to trigger tumor growth and the inflammatory response. The pan-bromodomain and extra-terminal domain (BET) inhibitor, (+)-JQ1 (**1**), was reported to inhibit angiogenesis. However, owing to the non-selectivity action of (+)-JQ1 towards all BET family members, the role of BRD4 and that of its bromodomains (BD1 and BD2) in angiogenesis remains elusive. Herein, we identified a potent BRD4 inhibitor, ZL0513 (**7**), which exhibited significant anti-angiogenic effects in chick embryo chorioallantoic membrane (CAM) and yolk sac membrane (YSM) models. This inhibitor also directly suppressed the viability and tube formation of human umbilical vascular endothelial cells (HUVECs). Moreover, ZL0513 (**7**) was found to inhibit the phosphorylation of c-jun and c-fos, important members of activating protein-1 (AP-1) transcription factor complexes that enhance angiogenesis. The findings on this novel BRD4 inhibitor indicate that, in addition to being a powerful pharmacological tool for further elucidating the roles and functions of BRD4 and its BD domains in angiogenesis, it may serve as a potential therapeutic strategy for targeting the vasculature in various angiogenesis-dysregulated human diseases.

## Introduction

The family of bromodomain and extra-terminal domain (BET) proteins is composed of bromodomain-containing protein 2 (BRD2), BRD3, BRD4, and bromodomain testis-specific protein (BRDT). BRD4, an epigenetic reader, is one of the most important BET proteins and plays an important role in angiogenesis and the development of inflammation-associated diseases, cardiovascular diseases, central nervous system disorders and cancers (Belkina and Denis, 2012; Shi et al., 2014; Padmanabhan et al., 2016; Tian et al., 2016; Wu et al., 2017; Liu et al., 2018; Tan et al., 2018; Tian et al., 2018; Tian et al., 2019a; Tian et al., 2019b; Zhao et al., 2019). Similar to other BET family members, BRD4 contains two highly conserved *N*-terminal bromodomains (BDs), BD1 and BD2, which can recognize acetylated lysine residues and non-histone proteins to transcriptionally regulate gene expression, cell cycle progression, cell proliferation, and apoptosis (Dey et al., 2000; Kanno et al., 2004; Dey et al., 2009). The sequences of BD1 and BD2 are highly conserved and homologous. However, each BD of BRD4 contributes to protein-specific binding. The two BDs of BRD4 may possess differential biological functions due to their interaction with different proteins. BRD4 BD1 shows higher affinity than BD2 for the tetra-acetylated histone H4 peptide. BRD4 BD2 recognizes and binds to diacetylated histone H3 and recruits non-histone proteins (Filippakopoulos et al., 2012; Shi et al., 2014; Liu et al., 2017). BRD4 is considered a promising therapeutic target in various human diseases (Filippakopoulos and Knapp, 2014; Cully, 2017; Liu et al., 2017; Cochran et al., 2019; Niu et al., 2019; Tian et al., 2019a; Tian et al., 2019b; Zhao et al., 2019; Alamer et al., 2020; Brasier and Zhou, 2020). Consequently, discovery and development of BRD4 inhibitors is urgently needed and has attracted increasing attention.

Currently, several BET inhibitors have been assessed in human clinical trials (Andrieu et al., 2016; Liu et al., 2017). One widely explored class of these small molecular inhibitors is azepine analogs, such as (+)-JQ1. (+)-JQ1 is one of the first discovered pan-BET family member inhibitor with nanomolar IC_50_ values (Filippakopoulos et al., 2010). Therefore, (+)-JQ1 has been extensively used as a powerful pharmacological tool to study the biological functions of the BET family in various diseases, such as leukemia, osteosarcoma, gallbladder cancer, colon cancer, and tumor angiogenesis (Zuber et al., 2011; Hu et al., 2015; Lee et al., 2015; Bid and Kerk, 2016; Bid et al., 2016; Hao et al., 2017). However, the applications of BET inhibitors have been limited due to their poor selectivity among BET family members. It has been revealed that BDs of the BET family function as important therapeutic targets for cancer, neurological disorders, inflammation, and obesity (Belkina and Denis, 2012; Padmanabhan et al., 2016). Therefore, some additional chemotypes of BET inhibitors have been designed to specifically target the BDs of BET family members, such as RVX-208, MS436, MS765, and Olinone. For example, RVX-208 and MS765 selectively bind to BD2 over BD1 of BET proteins, although they lack of selectivity towards BRD4 compared to other BRD family members (Chiang, 2014; Liu et al., 2018). Olinone is a BD1-selective inhibitor of BET family members that promotes the differentiation of oligodendrocyte progenitors (Chiang, 2014). The selectivity of small molecular inhibitors for BET BDs enables the study of the BET BD1- and BD2-specific functions in various diseases; however, these BD-specific inhibitors do not have a preference among BET family members. Accordingly, the development of more potent and specific BRD4 BD1- and BRD4 BD2-selective compounds with lower affinity for other BET family members is urgently needed for the further exploration of the specific roles and biological functions of each BRD4 BDs in various human diseases.

Angiogenesis plays a key role in a variety of pathological processes, including cancer, cardiovascular disease, inflammation, and obesity (Carmeliet and Jain, 2000; Carmeliet, 2003; Carmeliet, 2005; Srivastava et al., 2018). It is a multi-step process that relies on endothelial cell proliferation, migration to surrounding tissues and differentiation into new capillaries. BRD4 can broadly promote RNA polymerase II (RNAPII) pause release and drive angiogenesis (Chen et al., 2017). In addition, the bromodomain BET inhibitor (+)-JQ1 can inhibit vascular endothelial growth factor (VEGF)-induced angiogenesis by blocking vascular endothelial growth factor receptor 2 (VEGFR2) -mediated activation of p21 (RAC1) activated kinase 1 (PAK1) and endothelial nitric-oxide synthase (eNOS), inhibiting c-Myc and inactivating AP-1 (Baudino et al., 2002; Bid et al., 2016; Huang et al., 2016). However, which specific bromodomain contributes to BRD4-mediated angiogenesis remains unclear due to the non-selective pan-BET inhibitory activity of (+)-JQ1.

Through the screening of our in-house compound library targeting BET family proteins (Liu et al., 2018; Tian et al., 2018; Liu et al., 2020), we have discovered a potent BRD4 inhibitor, ZL0513 (**7**), that exhibits significant anti-angiogenic effect in chick embryo chorioallantoic membrane (CAM) and yolk sac membrane (YSM) models. This inhibitor can also directly suppress the viability and tube formation of human umbilical vascular endothelial cells (HUVECs). Herein, we also report the mechanistic findings of this inhibitor to suppress angiogenesis by regulating the phosphorylation of AP-1 transcription factors c-jun and c-fos.

## Materials and Methods

### Chemicals and Reagents

The potent pan-BET inhibitor, (+)-JQ1 (**1**), was purchased from Sigma-Aldrich **(**St. Louis, MO, USA). The stereoisomer of active (+)-JQ1, (−)-JQ1, which fails to significantly interact with any bromodomain of BET family members and was purchased from MedChemExpress (MCE, Monmouth Junction, NJ, USA) as a negative control. Each compound was dissolved in dimethylsulfoxide (DMSO, Sigma-Aldrich St. Louis, MO, USA) at a concentration of 20 mg/ml and maintained at −80°C. All the compounds of the in-house chemical library targeting BET family members were designed and synthesized by Dr. Zhou’s laboratory at the University of Texas Medical Branch (UTMB) following previously reported protocols (Liu et al., 2018; Tian et al., 2018; Niu et al., 2019; Liu et al., 2020), and the compounds were delivered for biological testing under the mutually signed material transfer agreement (MTA) with approval from the Office of Technology Transfer (OTT) of the UTMB. ZL0513 (**7**) was synthesized according to the detailed procedures described in the patent WO2018112037 A1, filed by the UTMB OTT, and a recent publication (Liu et al., 2020). The purity of all the tested compounds was higher than 95%, and for example, the purity of two highlighted BRD4 inhibitors, ZL0454 (**2**) and ZL0513 (**7**), was 98.0 and 97.3%, respectively, which was determined using HPLC analysis (**Supplementary Figures 1A, B**). Cell Counting Kit-8 (CCK8) was obtained from Beyotime (China). Rabbit anti-c-jun (abs131664), rabbit anti-phospho-c-jun (abs130717) and rabbit anti-c-fos (abs131453), and rabbit anti-phospho-c-fos (abs140099) were obtained from Absin (Shanghai, China).

### Cell Line

Human umbilical vein vascular endothelial cells (HUVECs, PCS-100-010) were obtained from American Type Culture Collection (ATCC, Manassas, VA, USA). The cells were grown in endothelial cell growth medium supplemented with growth supplements (EGM, CC-3124, Lonza) and cultured at 37°C in a humidified incubator supplemented with 5% CO_2_.

### Chick Embryo Chorioallantoic Membrane (CAM) Assay

Fertilized white leghorn chicken eggs were obtained from the Avian Farm of South China Agriculture University (Guangzhou, China) and the shells were cleaned with 1% geramine. The eggs were incubated under 50–60% humidity at 37.5°C. A small window (10 × 10 mm^2^) was cut above the air chamber of egg while the chick embryo was growing through the ninth day. BET family member inhibitors or DMSO (dissolved in 30 µl of phosphate-buffered saline [PBS, pH 7.4]) were placed directly onto the live chick embryo CAM through the window at the indicated concentrations and incubated for 48 h. Then, the eggshells were cut carefully along the axis of the median line, and the content was discarded. The CAM vasculature around the eggshell windows was observed and photographed under a stereomicroscope (Olympus SZX16). The microvessel density (MVD) was calculated as the percentage of blood vessel area in the total drug-treated area using the Image-Pro Plus 6.0 software (IPP 6.0, Media Cybernetics, Inc., Rockville MD, USA).

### Chick Embryo Yolk Sac Membrane (YSM) Assay

On the third day of incubation, the well-developed live chicken embryos were carefully transferred into sterilized dishes, and the chick embryo vessels were arranged to face upward. Color marked silastic rings were symmetrically placed onto the top of the vessel regions of the yolk sac membrane and incubated for 3 h in the incubator. Then, 100 μM BET family member inhibitors or DMSO (dissolved in 30 µl of PBS, pH 7.4) was added into the center of the rings and cultured in the incubator for 24 h. Images of the vessels in the silastic rings were captured at 0 and 24 h, respectively. The images were quantitatively analyzed by Image-Pro Plus 6.0, and then the number of blood vessels 24 h after drug treatment and the MVD at 0 and 24 h were quantified using IPP 6.0. The growth rate in the DMSO-treated silica gel circle was considered to be one.

### Time Resolved-Fluorescence Resonance Energy Transfer (TR-FRET) Assays and IC_50_ Measurements

The binding affinities and IC_50_ values of the tested BET family member inhibitors for the BRD4 and BRD2 proteins bromodomains (BDs) were detected using a commercially available TR-FRET assay kits (Cayman Chemical, Ann Arbor, MI, USA) according to the manufacturer’s instructions and our recent publications (Borysko et al., 2018; Liu et al., 2018; Liu et al., 2020). The specificity of the designed and synthesized compounds for BRD4 was also evaluated through comparison with those for BRD2.

### Cell Viability Evaluation by CCK-8 Assay

Cell viability after BET family member inhibitor treatment was evaluated by CCK-8 assay. HUVECs were seeded in 96-well plates at a density of 2,000 cells/well. Twenty-four hours later, the BET family member inhibitors, (−)-JQ1 (negative control drug) and DMSO (solvent group) were added at the indicated concentrations. Forty-eight hours after treatment, 10 μl of CCK-8 reagent was added to each well and incubated at 37°C for an additional 3 h. The absorbance was evaluated at 450 nm to determine cell viability.

### Tube Formation Assay

The HUVECs treated with 8.5 μM ZL0513 (**7**), (+)-JQ-1 (**1**), (−)-JQ1, or DMSO for 48 h were harvested and seeded in a Matrigel-precoated 96-well plate at a density of 3 × 10^4^ cells/well. At the same time, the cells were also treated with the same dose of ZL0513 (**7**), (+)-JQ-1 (**1**), (−)-JQ1, or DMSO in endothelial cell growth medium without growth supplements. Then, the cells were maintained at 37°C for an additional 5 h to observe the inhibitory effects of ZL0513 (**7**), (+)-JQ-1 (**1**), (−)-JQ1, and DMSO on the angiogenic ability of vascular endothelial cells. The circumference of the formed tubes was measured under an inverted light microscope.

### Immunoblot Assays

HUVECs were treated with DMSO, (−)-JQ1, (+)-JQ1 (**1**), or ZL0513 (**7**) for 48 h. Then, the proteins of these cells were extracted using RIPA buffer and separated by 10% (w/v) sodium dodecyl sulphate-polyacrylamide gel electrophoresis (SDS-PAGE). The separated proteins were transferred onto a polyvinylidene fluoride (PVDF) membrane (Millipore, MA, USA) and blocked using 5% (w/v) non-fat powder milk at room temperature. Next, the membranes were incubated in the relative primary antibody and horseradish peroxidase (HRP)-conjugated secondary antibodies. Then, the Image Quant LAS 4000 system (GE Healthcare, Waukesha, WI, USA) was used for the direct visualization of the membranes. The protein bands were densitometrically quantified using Quantity-One protein analysis software (Bio-Rad Laboratories, Hercules, CA, USA). The relative expression of the proteins was normalized to the expression of glyceraldehyde-3-phosphate dehydrogenase (GAPDH).

### Statistical Analysis

All the data are presented as the means ± standard deviation (SD) from three independent experiments. Student’s *t*-test was used to determine the differences between two groups. *P* values < 0.05 (*P* < 0.05) were considered to indicate a significant difference.

## Results

### Evaluation of the Anti-Angiogenic Effects of New BET Family Member Inhibitors in a CAM Model

Given that a number of small molecular BET inhibitors and their cocrystal structures with BET family members are available, we have designed and synthesized an in-house chemical library by targeting BET family proteins through structure-based drug design, fragment-based drug design, and computer-aided drug design (Liu et al., 2018; Niu et al., 2019; Liu et al., 2020). The preliminary results of 16 selected compounds are presented in **Table 1**, including the commercially available BET family member selective inhibitors (+)-JQ1 (**1**), ZL0454 (**2**), and MS436 (**3**), as well as other representative compounds that are selected from a primary assay used to explore anti-angiogenesis through functional studies. Specifically, the (+)-JQ1, a widely used BET family member inhibitor, was employed as the positive control for comparison.

**Table 1:** Screening for the anti-angiogenic activity of synthesized BET inhibitors using the chick embryo CAM model.

Compounds	1	2	3	4	5	6	Positive Ratio
Egg number							
DMSO	+	+	+	-			
(+)-JQ1(1)	+++	+++	++	++	+	++	5/6
ZL0454(2)	+++	++	+++	++	+	+++	5/6
MS436(3)	++	+++	+++	+++	+		4/5
ZL0164(4)	-	-	++	++	+		2/5
ZL0452(5)	-	+	++	+			1/4
ZL0453(6)	+	-	++	+	+		1/5
ZL0513(7)	++	++	+++	+	++		4/5
ZL0518(8)	++	-	-	-	++	+	1/3
ZL0519(9)	++	-	++	+++			3/4
ZL0520(10)	+	-	++	+	-		1/5
ZL0549(11)	++	-	++	+++			3/4
ZL0586(12)	++	+	+	++	+		2/5
ZL0588(13)	-	++	++	-	+		2/5
ZL0589(14)	++	-	-	++	+		2/5
ZL0593(15)	++	++	+	++	-		3/5
HJC5100(16)	+	-	+				-

**Figure 1:**
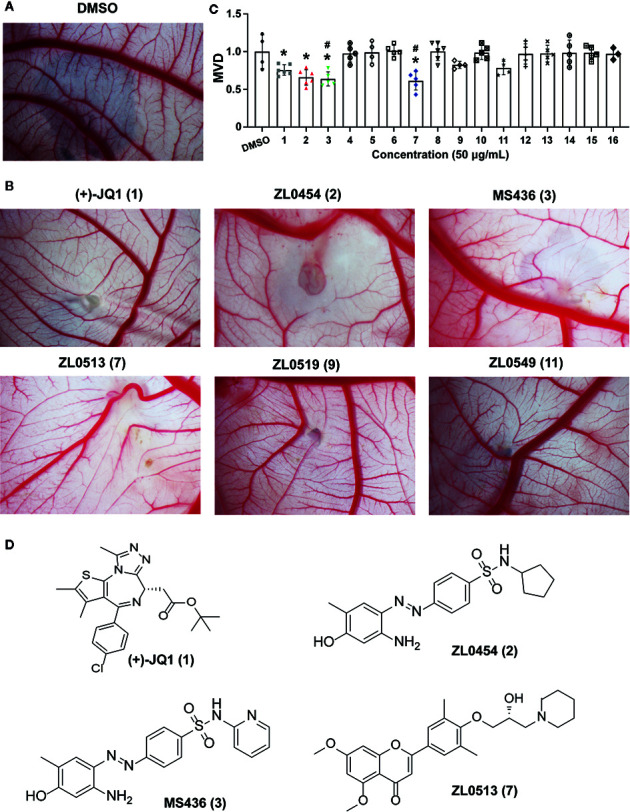
Initial screening of select BET inhibitors to determine their effects on angiogenesis in the chick embryo CAM model. Representative inhibitory results on the growth of blood vessel branches by DMSO **(A)** or the selected BRD4 inhibitors **(B)**, which showed strong positive inhibition (+++) of angiogenesis in the chick embryo CAM model. Each BET inhibitors (50 μg/ml) or DMSO was added directly onto the live 9-day-old chick embryo CAM model and incubated for 48 h. Then, the blood vessel network in the chicken embryo CAM was photographed by an OPTPRO 2007 image system. **(C)** The statistical results of microvessel density (MVD) analysis of the chick embryo CAM model treated with the selected BET inhibitors are shown in **Table 1**. **(D)** Structures of (+)-JQ1 (1) (positive control), ZL0454 (2), MS436 (3), and ZL0513 (7). *P < 0.05 compared with the DMSO group, ^#^ *P* < 0.05 compared with the (+)-JQ1 (1) positive control group.

The effects of all these selected compounds on angiogenesis were first determined through use of the chick embryo CAM model. The CAM assay is a well-established model for screening anti-angiogenic agents, because the ability of compounds to prevent blood vessel formation can be easily detected (Deryugina and Quigley, 2008; Chen et al., 2010). Each of the compounds (50 μg/ml) was added to the CAM of 9-day-old chick embryos for 48 h. The CAMs of chick embryos were photographed, and the inhibition ratio indicating angiogenesis was calculated and analyzed. The anti-angiogenic efficacy of these compounds was classified as negligible to strong, as indicated in **Supplementary Figure 1C**. All the inhibitory rates of these compounds were first calculated as the number of chick embryos with greatly inhibited vasculature formation (++ and +++) compared with the total number of experimental chick embryos. An inhibitory effect observable but not obvious on the generation of third-grade vessels was indicated by “+”; an obvious medium-level inhibitory effect on secondary vessels was indicated by “++”; and an obvious potent inhibitory effect on primary blood vessels was indicated by “+++”. As shown in **Table 1**, (+)-JQ1 (**1**), ZL0454 (**2**), MS436 (**3**), ZL0513 (**7)**, ZL0519 (**9**), and ZL0549 (**11**) showed the strongest positive inhibition (+++) of angiogenesis among all the selected compounds in the library. Representative photographs of CAMs after treatment with DMSO or strong inhibitory drugs are shown in **Figures 1A, B**.

Then, the anti-angiogenic impact on the growth of the blood vessel branch by the selected BET inhibitors in the chick embryo CAM model was quantified using IPP software. The statistical analysis showed that, among these selective compounds, (+)-JQ1 (**1**), ZL0454 (**2**), MS463 (**3**), and ZL0513 (**7)** exhibited the most impressive inhibitory effect on MVD (**Figure 1C**). Furthermore, the inhibitory effect of MS463 (**3**) and ZL0513 (**7)** on MVD was better than that of the (+)-JQ1 positive control. The structures of all these compounds are shown in **Figure 1D** and **Supplementary Figure 2**.

### ZL0513 Displays Anti-Angiogenic Effects in a Concentration-Dependent Manner in a Chick Embryo CAM Model

**Figure 2:**
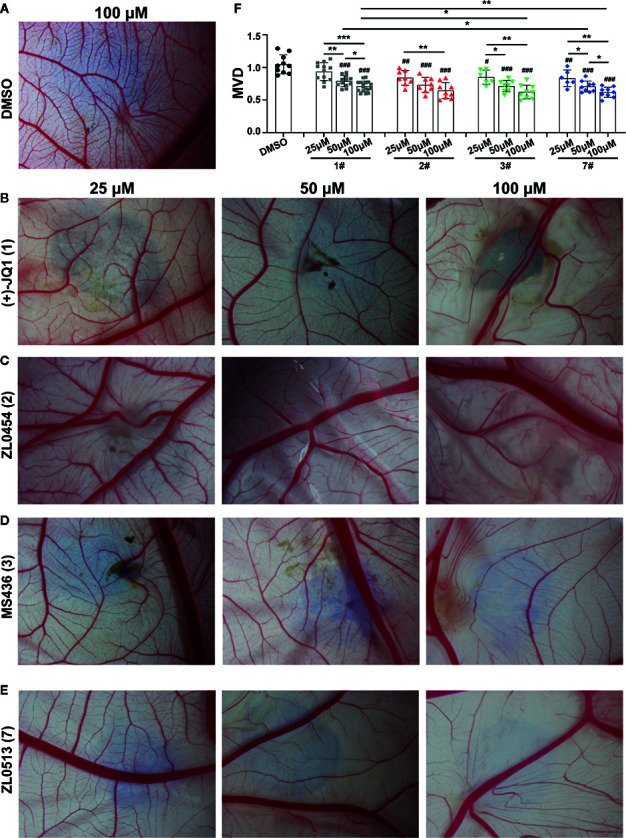
ZL0513 shows anti-angiogenic activity in a concentration-dependent manner in the chick embryo CAM model. Representative images of the growth of blood vessel branches in the chick embryo CAM model. DMSO (negative control, **A**), (+)-JQ1 (**1**) **(B)**, ZL0454 (2) **(C)**, MS436 (3) **(D)**, and ZL0513 (7) **(E)** are presented. Each of these compounds (25, 50, and 100 μM) or DMSO was added directly onto the live 9-day-old chick embryo CAM model and incubated for another 48 h. Then, the blood vessel network in the chicken embryo CAM was photographed. **(F)** The statistical analysis of the MVD in the chick embryo CAM model, as described above (n ≥ 6 of each group). Except for the 25 μM (+)-JQ1 (**1**) treatment, all inhibitor treatments at all concentrations significantly reduced the MVD in the chick embryo CAM model compared with the effect of DMSO. ^#^ *P* < 0.05, ^##^ *P* < 0.01, and ^###^ *P* < 0.001 compared with the DMSO group. Furthermore, the anti-angiogenic activity of (+)-JQ1 (**1**) and ZL0513 (**7**) was shown in a concentration-dependent manner and the inhibitory rate of ZL0513 (**7**) was higher than that of (+)-JQ1 (1). *P < 0.05, **P < 0.01, and ***P < 0.001.

We confirmed the angiogenic inhibition efficacy of ZL0454 (**2**), MS463 (**3**), and ZL0513 (**7)** applied at different concentrations. Compounds of 25, 50, and 100 μM were added to the CAM of 9-day-old chick embryos and incubated for 48 h, and then, the CAMs were photographed for further analysis of the anti-angiogenic drug efficacy. The results showed a dense capillary plexus and multiple tiny capillaries originating from terminal capillaries in the DMSO group (**Figure 2A**). However, the reduction in the main blood vessel branches of CAM blood vessels at the site of drug administration was notable in the groups treated with (+)-JQ1, ZL0454 (**2**), MS463 (**3**), or ZL0513 (**7)** compared with that of the group treated with DMSO (**Figures 2B–E**). The statistical analysis results demonstrated that, compared to DMSO, (+)-JQ1, ZL0454 (**1**), MS463 (**2**), and ZL0513 (**7**) significantly inhibited MVD in a concentration-dependent manner (**Figure 2F**). Nevertheless, ZL0454 (**1**), MS463 (**2**), and ZL0513 (**7**) exhibited stronger inhibitory efficacy than (+)-JQ1 on MVD at each of the treated concentrations.

As acetylated histone peptides are known substrates for bromodomain-containing proteins, we initially screened the complexes for their ability to modulate the protein–protein interaction between acetylated lysine and BET family members. The BRD4 selectivity profile of (+)-JQ1 (**1),** ZL0454 (**2**), MS463 (**3**), and ZL0513 (**7)** was also evaluated through a comparison with that of its close family member BRD2. Then, the IC_50_ of the compounds that prevent BRD4 (BD1 or BD2)/BRD2 (BD1 or BD2) and acetylated histone interactions was calculated by TR-FRET assay *in vitro*. As shown in **Table 2**, (+)-JQ1 possessed potent inhibitory activity towards all BET family proteins with no selectivity for any individual member. Importantly, (+)-JQ1 also showed no preference between BD1 and BD2 among the BET family proteins. In contrast, ZL0454 (**1**), MS436 (**2**), and ZL0513 (**7)** exhibited significant inhibitory activity towards BRD4 over BRD2. In addition, ZL0454 (**1**) showed no preference between BD1 and BD2, as previously reported (Liu et al., 2018). Similar to MS436 (**2**), ZL0513 (**7**) exhibited a potent preference towards BRD4 BD1 over BRD4 BD2. Moreover, the IC_50_ value of ZL0513 (**7**) was higher than that of MS436 (**2**). As presented in our recently published report, ZL0513 (**7**) exhibits a potent preference towards BRD4 BD1 but not the other BET family members or the BD2 domain (Liu et al., 2020). Collectively, the data indicated that ZL0513 (**7**) may be a new potent BRD4 inhibitor for use as an orally bioavailable pharmacological tool to further explore the biological functions of the BD1 domain of BRD4.

**Table 2:** Binding affinities of (+)-JQ1, ZL0454, MS436, and ZL0513 for BRD4 and the selectivity over BRD2 based on TR-FRET assays.

	BRD4 BD1 (IC_50_, nM)	BRD4 BD2 (IC_50_, nM)	BRD2 BD1 (IC_50_, nM)	BRD2 BD2 (IC_50_, nM)
(+)-JQ1	72	62	78	52
ZL0454 (4)	49	54	772	1,836
MS436 (5)	73	791	797	846
ZL0513 (7)	67	684	791	845

### ZL0513 Suppresses New Blood Vessel Formation in a Chick Embryo YSM Model

**Figure 3:**
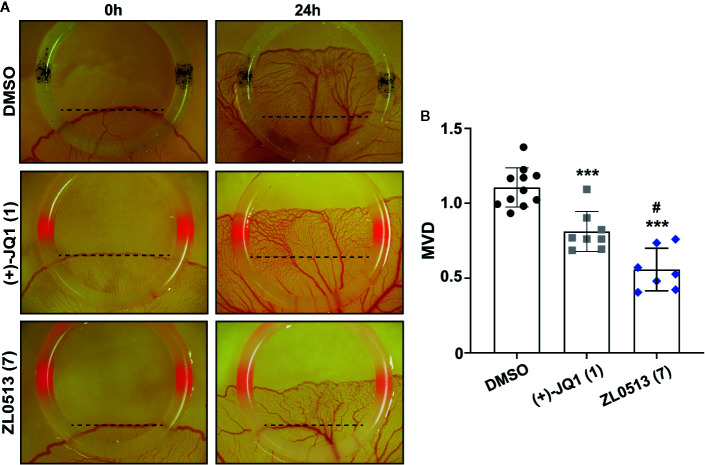
*In vivo* anti-angiogenic activity assay of ZL0513 using a chick embryo YSM model. **(A)** Representative images of the vascular network in the chick embryo YSM model treated with DMSO, (+)-JQ1 (**1**), and ZL0513 (**7**) at 100 μM. Drugs were administered on the vascular plexus of the YSM inside a rubber ring, which was marked black in the DMSO treatment group and marked red in the drug treatment groups. The YSMs were photographed at 0 and 24 h after drug treatment using an OPTPRO 2007 image system. **(B)** MVD was analyzed using Image-Pro Plus 6.0 image analytical software. Statistical analysis showed that (+)-JQ1 (positive control) and ZL0513 (7) significantly reduced MVD in the YSM model compared with the effect of DMSO. ***P < 0.001 compared with the DMSO group; ^#^ *P <* 0.01 compared with the (+)-JQ1 group.

The chick embryo YSM model, another well-developed model for screening anti-angiogenic drugs, was employed to confirm the anti-angiogenic role of ZL0513 (**7)** because of its potent inhibitory effect against angiogenesis compared with positive control groups. Furthermore, ZL0513 (**7**) was selected for further analysis of the anti-angiogenic role of the BRD4, and (+)-JQ1 (**1)** was used as a reference compound for comparison. ZL0513 (**7)** and (+)-JQ1 (**1)** was added at 100 μM to the YSM of 3-day-old chick embryos, which were then photographed at the indicated times for further analysis of the anti-angiogenic drug capabilities. As shown in **Figure 3A**, our results implied that the capillary plexus in the chick embryo YSM of the DMSO-treated group was dense and resembled a honeycomb network of tiny capillaries. However, the slow growth of blood vessels and reduced number of tiny capillaries were observed in the chick embryo YSM treated with (+)-JQ1 (**1)** and ZL0513 (**7**). The statistical analysis showed that the blood vessel density and the number of blood vessels were significantly reduced after 24-h treatment with (+)-JQ1 (**1)** and ZL0513 (**7**) compared with that after DMSO treatment (**Figure 3B**). Moreover, the ZL0513 (**7**) inhibition efficacy on angiogenesis in the chick embryo YSM was significantly higher than that of (+)-JQ1 (**1)** treatment.

### ZL0513 Inhibits the Viability and Tube Formation of HUVECs by Regulating AP-1 Expression *In Vitro*

**Figure 4:**
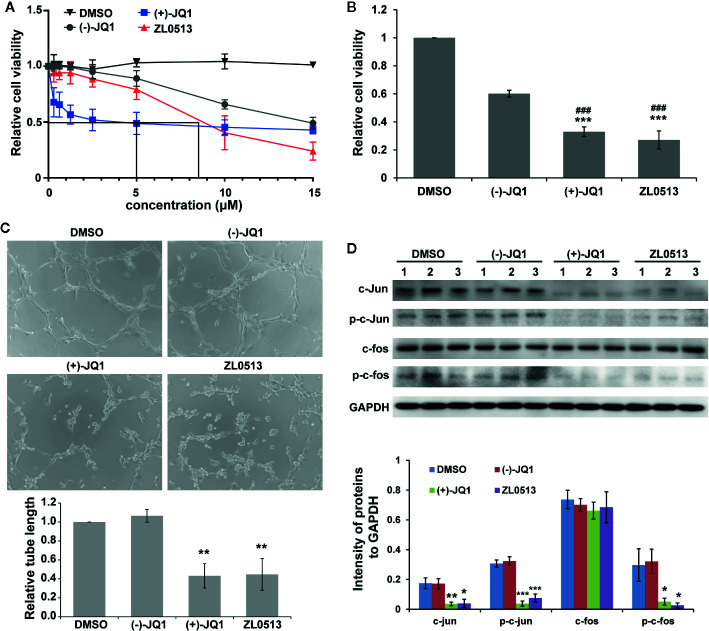
ZL0513 inhibits the viability and tube formation of HUVECs by inactivating AP-1 expression. **(A)** Relative cell viability of the HUVECs treated with the indicated concentrations of DMSO (solvent control), (−)-JQ1 (negative control), (+)-JQ1 (**1**), or ZL0513 (7). **(B)** Relative viability of the HUVECs was determined using CCK8 assays after 8.5 μM (−)-JQ1, (+)-JQ1 (**1**), or ZL0513 (7) treatment for 96 h. Both (+)-JQ1 (**1**) and ZL0513 (**7**) significantly inhibited cell viability compared with DMSO or (−)-JQ1. **(C)** Representative HUVEC tube formation after treatment with DMSO, (−)-JQ1, (+)-JQ1 (**1**), or ZL0513 (**7**). HUVECs, which were pretreated with DMSO, (−)-JQ1, (+)-JQ1 (**1**), or ZL0513 (**7**) for 48 h, were harvested and seeded in Matrigel. Each figure is representative of at least three independent repeats. Statistical analysis of the length of the tube showed that (+)-JQ1 (**1**) and ZL0513 (**7**), but not (−)-JQ1, significantly inhibited tube length of the HUVECs compared with the effect of DMSO. **(D)** The expression of AP-1 components in the HUVECs treated with DMSO, (−)-JQ1, (+)-JQ1 (**1**), or ZL0513 (**7**). The expression of total proteins and phosphorylation of c-jun and c-fos were significantly decreased in the HUVECs treated with (+)-JQ1 (**1**) or ZL0513 (7) at the same dose. *P < 0.05, **P < 0.01 and ***P < 0.001 that compared with the DMSO group; ###*P* < 0.001 compared with the (−)-JQ1 group. Scale bars: 50 μm.

A previous report indicated that JQ1 significantly inhibited the viability of HUVECs in a concentration-dependent manner (Bid et al., 2016). Therefore, we investigated whether ZL0513 (**7**) possesses an inhibitory effect similar to that of JQ1 on the viability of HUVECs, focusing on a drug concentration range from 0.3 to 15 μM. Furthermore, (−)-JQ1 was employed as a negative control, and DMSO was employed as a solvent control. ZL0513 (**7**), similar to (+)-JQ1 (**1**), could inhibit cell viability in a concentration-dependent manner with the 50% inhibition at 8.5 μM (**Figure 4A**). In addition, (+)-JQ1 (**1**) suppressed cell viability at a slightly lower 50% inhibition concentration (5 μM) than that of ZL0513 (**7**). Applied as 96-h posttreatments at a concentration of 8.5 μM, (+)-JQ1 (**1**) and ZL0513 (**7**) each significantly suppressed cell viability compared with the effect of DMSO or (−)-JQ1 in HUVECs (**Figure 4B**).

In addition, we examined the anti-angiogenic role of ZL0513 (**7**) *in vitro* through a tube formation assay. HUVECs were seeded on Matrigel, and then treated with (+)-JQ1 (**1**) and ZL0513 (**7**) at 8.5 μM and allowed to form tubes. As shown in **Figure 4C**, the circumference of the tubes formed by cells treated with (+)-JQ1 (**1**) or ZL0513 (**7**) was smaller than that of the tubes treated with DMSO or (−)-JQ1. The statistical analysis showed that a reduction in tube circumference was observed in both the (+)-JQ1 (**1**)- and ZL0513 (**7**)-treated groups, compared with that in the cells treated with DMSO. In addition, (−)-JQ1 had no inhibitory effect on tube formation compared with that induced by DMSO. However, the length of tubes was not significantly different in the (+)-JQ1 (**1**)- and ZL0513 (**7**)-treated groups.

A previous report indicated that (+)-JQ1 (**1**) inhibited AP-1 activity and further decreased the expression of the AP-1 component FOSL1 in HUVECs (Bid et al., 2016). Therefore, we sought to determine whether (+)-JQ1 (**1**) and ZL0513 (**7**) could inhibit the expression of c-jun and c-fos, other components of AP-1. The results showed that, compared with DMSO, both (+)-JQ1 (**1**) and ZL0513 (**7**) significantly inhibited the expression of c-jun, but not c-fos, and the phosphorylation of both c-jun and c-fos (**Figure 4D**). In addition, compared with DMSO, (−)-JQ1 displayed no inhibitory effect on the components of AP-1 in the HUVECs.

## Discussion

Angiogenesis is a vital process through which vascular endothelial cells sprout from pre-existing vessels and form capillaries during cell growth and development that and supply oxygen and nutrients to every cell in the body under physiological and pathological stress conditions. Recent studies indicate that angiogenesis is dysregulated in various pathological processes, leading to various diseases, including cancer, cardiovascular disease, inflammation, and obesity (Carmeliet and Jain, 2000; Carmeliet, 2003; Srivastava et al., 2018). It has been established that the abnormal formation of blood vessels is effective as a treatment for patients with cancer, chronic liver disease, cardiovascular disease or other diseases; therefore, using anti-angiogenic agents as inhibitors has become a major therapeutic strategy (Liao et al., 2014; Chen et al., 2018; Srivastava et al., 2018). Currently, several angiogenesis inhibitors that specifically target VEGF, VEGFR, or other specific genes that are involved in angiogenesis can prolong life expectancy in patients with advanced colon cancer and are now being investigated in clinical trials alone or in combination with conventional therapeutic approaches of both cancer and retinal disease (Hurwitz et al., 2004; Avery et al., 2006). However, clinical side effects of anti-VEGF/VEGFR therapy have begun to emerge, thus showing less benefit in some types of diseases (Sitohy et al., 2012). Therefore, the design and synthesis of more target-specific and effective anti-angiogenic agents for disease therapies have remained imperative in recent years. The pan-BET bromodomain inhibitor (+)-JQ1 has been identified as capable of significantly suppressing tumor angiogenesis (Huang et al., 2016). In our work, we designed a series of specific inhibitors of BET family members through structure-based drug design. By screening drug candidates through chick embryo models, we have demonstrated that BRD4 can serve as a druggable angiogenesis therapeutic target.

BET family members (BRD2, BRD3, BRD4, and BRDT) contain two highly conserved bromodomains (BD1 and BD2), an ET domain, and a C-terminal domain. Disrupting the interactions between BET bromodomains and acetylated lysine residues is one of the most drug-induced therapeutic approaches and can effectively inhibit cancer and inflammation (Sorkin and Von Zastrow, 2002; Belkina and Denis, 2012; Vidler et al., 2012; Filippakopoulos and Knapp, 2014; Romero et al., 2016; Liu et al., 2018). Therefore, the discovery and development of selective BRD4 BD1 and BD2 inhibitors among BET family proteins have attracted increasing attention in recent years for use in studying their unique functions. It has been reported that JQ1 can induce insulin production in pancreatic β cells. However, the specific inhibition of BRD4 significantly enhances insulin transcription and increases the insulin content level, while specific BRD2 inhibition increases fatty acid oxidation in pancreatic β cells (Deeney et al., 2016). It was also suggested that BET proteins possess specific functions at different developmental stages and/or in different cell types in mouse testes (Filippakopoulos and Knapp, 2014). Most of the currently available BET inhibitors, such as (+)-JQ1, are pan-BET bromodomain inhibitors, lack selectivity for BET family members and impact multiple regulatory pathways (Andrieu et al., 2016). Therefore, it is challenging to distinguish the exact functions of each BET protein using the currently available pan-BET bromodomain inhibitors. BRD4 is a member of the BET family and represents a promising therapeutic target for various types of diseases. ZL0513 (**7**) has a better selectivity of BRD4 over BRD2 at the similar level to that of ZL0580, another BRD4 inhibitor we recently reported (Niu et al., 2019). In our previously report, we demonstrated that BRD4-KO, but not BRD2-KO, can absolutely abolish the function of ZL0580 in J-lat cells (Niu et al., 2019). Furthermore, we reported the function of suppressing HIV with ZL0580, while pan-BET inhibitor (+)JQ-1 was reported to have a different function by activation of HIV latency instead (Alamer et al., 2020). Since the selectivity window to BRD4 is only 10-fold than that BRD2, an effect of BRD2 with ZL0513 may not be excluded. Herein, we demonstrated that BRD4 possesses a pro-angiogenic role. BRD4 inhibition by ZL0513(**7**) can markedly suppress angiogenesis.

The bromodomains BD1 and BD2 have been validated as epigenetic reader domains that selectively recognize acetylated lysine residues on the tails of histone proteins and non-histone proteins and are used to investigate pan-BET inhibitors in various diseases (Dhalluin et al., 1999; Borysko et al., 2018). Among all the BET proteins, BD1 and BD2 exhibit approximately 44% homology to each other (Nakamura et al., 2007). The sequences and structural differences suggest that BD1 and BD2 may have distinct functions under physiological or pathological conditions. It has been reported that BD1 can bind to the acetylated lysine residue of histone H3, thereby recruiting downstream proteins and initiating transcription (Shi et al., 2014). BD2 is mostly in a free state and is associated with the recruitment of non-histone proteins. BRD4 BD2 has been shown to interact with the cyclin T1 component of human positive transcription elongation factor b (P-TEFb), the RFC-140 subunit of human replication factor C (hRFC140), signal-induced proliferation-associated protein 1 (SPA-1), and the human papillomavirus type 11 (HPV-11) E2 protein (Borysko et al., 2018). Due to the lack of domain- and BET protein-specific inhibitors, the exact regulatory mechanisms of BRD4 BD1 and BRD4 BD2 remain to be elucidated. Over the past decade, researchers have focused on developing highly selective BRD4 inhibitors and domain-selective inhibitors. We previously reported that ZL0420 and ZL0454 (**2**), which are BRD4-selective but not BD1 and BD2 bromodomain- selective inhibitors, inhibited acute airway inflammation (Liu et al., 2018). Therefore, the development of new inhibitors that selectively distinguish BRD4 BD1 from BRD4 BD2 will present an opportunity to clarification of the regulatory function and pathway of each bromodomain of BRD4. Herein, we screened an in-house chemical library designed based on BET structures. Interestingly, we identified a novel BRD4 inhibitor, ZL0513 (**7**), which can significantly inhibit angiogenesis. In our recently published report, we demonstrated that ZL0513 (**7**) exhibited potent preference towards BRD4 BD1 but not the other BET family members or the BD2 domain, a finding unambiguously validated by co-crystal structure of the inhibitor in complex with the human BRD4 BD1 protein (Liu et al., 2020). Since BD1 can bind to the acetylated lysine residue of histone H3, the on-target effect of ZL0513 (**7**) was also determined by examining the level of H3K122Ac. ZL0513 (**7**) was also found to block poly(I:C)-induced H3K122Ac (Liu et al., 2020). In summary, ZL0513 (**7**) functions as a BRD4 inhibitor, and the BRD4 possesses pro-angiogenic capabilities and plays an important role in angiogenesis-related diseases.

AP-1 can mediate VEGF-induced HUVEC migration and proliferation (Jia et al., 2016). Among multiple transcription factors, AP-1 protein and activity levels were dramatically decreased by JQ1 in HUVECs (Bid et al., 2016). AP-1 transcription factor complexes consist of Jun family members (c-jun, JunB, and JunD) and Fos family members (c-fos, FOSL1, FOSL2, and FosB) (Chinenov and Kerppola, 2001; Hess et al., 2004; Jia et al., 2016). The transcriptional activity of AP-1 is specifically mediated by the phosphorylation of their family members. JQ1 can significantly inhibit AP-1 activity and FOSL1 expression in HUVECs (Bid et al., 2016). However, whether the inhibition of BRD4, especially the inhibition of BRD4 BD1, can suppress the expression and phosphorylation of other components of AP-1 is still unknown. To this end, we demonstrated that the inhibition of BRD4 BD1 can inhibit the expression of c-jun, but not c-fos, and the phosphorylation of c-jun and c-fos. Herein, we clarified that the BRD4 BD1 regulates the phosphorylation of c-jun.

In summary, we have identified a potent BRD4 inhibitor ZL0513 (**7**) that exhibit significant anti-angiogenic effects in CAM and YSM models. This inhibitor can also directly inhibit the viability and tube formation capability of HUVECs. Moreover, ZL0513 (**7**) was found to inhibit the phosphorylation of c-jun and c-fos, important members of AP-1 transcription factor complexes that enhance angiogenesis. The findings on this novel BRD4 inhibitor suggests that, in addition to being a powerful pharmacological tool for further elucidating the roles and functions of BRD4 and its BD domains in angiogenesis, it may also serve as a potential therapeutic strategy for targeting the vasculature in various angiogenesis-dysregulated human diseases.

## Data Availability Statement

All datasets generated for this study are included in the article/**Supplementary Material**.

## Ethics Statement

The animal study was reviewed and approved by Experimental Animal Ethics Committee of Guangdong Pharmaceutical University.

## Author Contributions

Participated in research design: Q-QZ, LW, RZ, and JZ. Conducted experiments: ZZ, ZL, LH, JZ, YY, LL, XL, JC, and Q-QZ. Contributed new reagents or analytic tool: ZL and JZ. Performed data analysis: ZZ, LL, LH, and ZL. Wrote or contributed to the writing of the manuscript: Q-QZ, ZZ, ZL, and JZ.

## Funding

This work was supported by National Natural Science Foundation of China (Grant numbers 31872833, 31771578), Natural Science Foundation of Guangdong Province (Grant number 2018A030313186), UTMB Technology Commercialization Program, Sanofi Innovation Awards (iAwards), Crohn’s & Colitis Foundation Entrepreneurial Investing (EI) Initiative award, Research Fellowship Award from the Crohn’s & Colitis Foundation of America (Grant number 548813), and Innovation and university promotion project of Guangdong Pharmaceutical University (Grant number 2017KCXTD020).

## Conflict of Interest

The authors declare that the research was conducted in the absence of any commercial or financial relationships that could be construed as a potential conflict of interest.
