# Supplementary material for: A Bromodomain-Containing Protein 4 (BRD4) Inhibitor Suppresses Angiogenesis by Regulating AP-1 Expression
Source: Front Pharmacol. 2020 Jul 10;11:1043. doi: 10.3389/fphar.2020.01043 (PMC7381267; doi:10.3389/fphar.2020.01043)
Supplement: Supplementary file 1 [file DataSheet_1.pdf]

Supplementary Material

1. Supplementary Figures

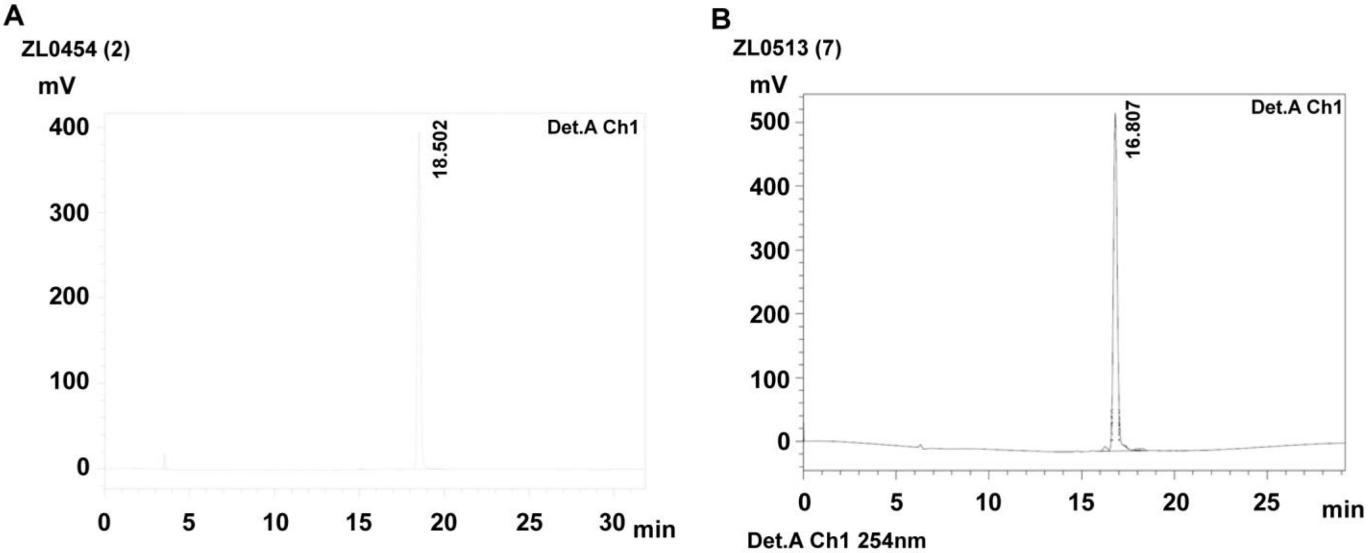

Det.A Ch1 254nm (A)

Peak#	Ret. Time	Area	Area%	Height%
1	3.526	89046	1.742	4.391
2	15.122	11526	0.226	0.240
3	18.502	5010308	98.032	95.369
Total		5110880	100.000	100.000

Det.A Ch1 254nm (B)

Peak#	Ret. Time	Area	Area%	Height%
1	16.28	114599	1.425	1.324
2	16.807	7758250	97.294	97.285
3	17.308	14540	0.182	0.212
4	17.988	40701	0.510	0.563
5	18.251	46945	0.589	0.615
Total		7974036	100.000	100.000

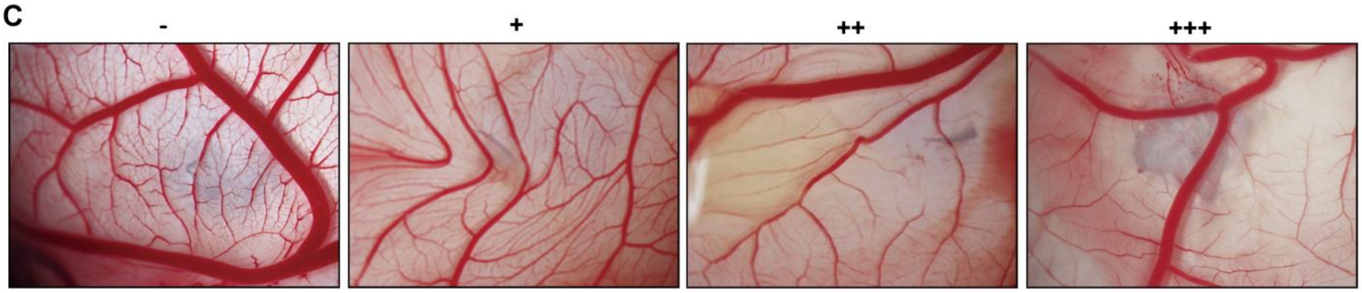

Supplementary Figure 1. The purity of two highlighted BRD4 inhibitors and diagram of inhibition level on angiogenesis in chick embryo CAM model. (**A**) ZL0454 (2) and ZL0513 (7) was detected using HPLC. HPLC spectra of (A) ZL0454 (2) and (B) ZL0513 (7). (**B**) Diagram of inhibition level on angiogenesis by BRD4 inhibitors in chick embryo CAM model. No apparent inhibition on blood vessels was marked as “-”. The inhibition of micro vessels formation was marked as “+”, “++” indicates an obvious inhibitory on the three-blood vessel network, and “+++” presents the significantly inhibition of secondary vessels branch.

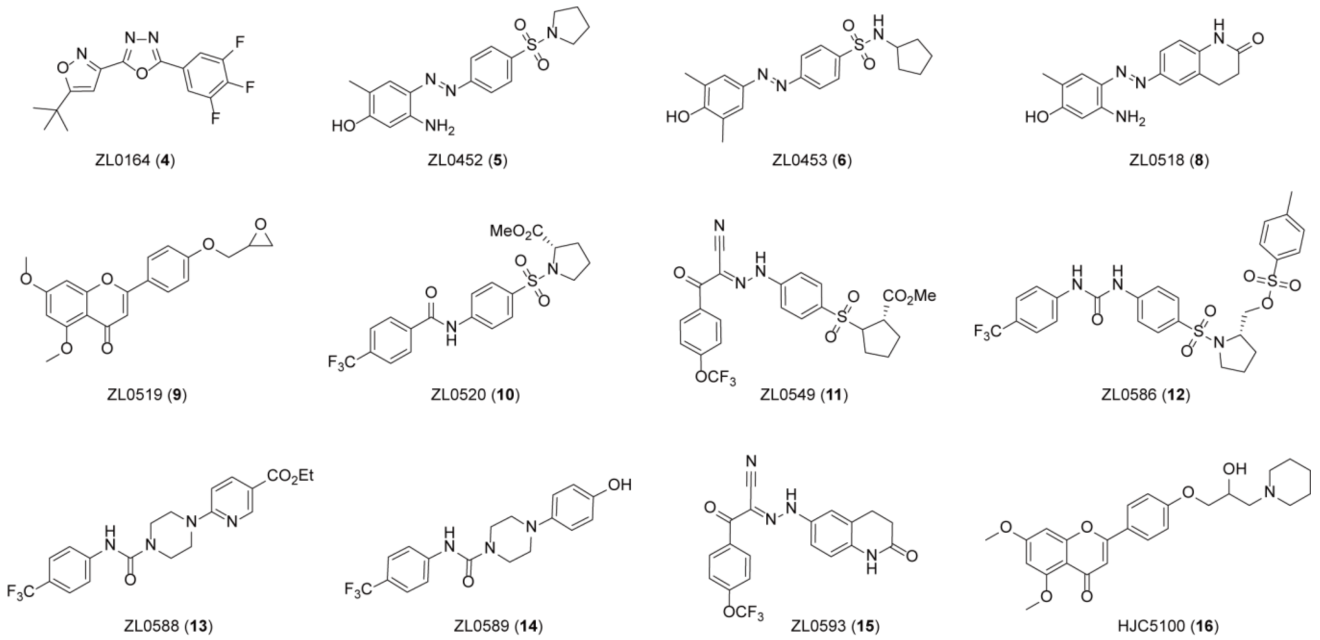

Supplementary Figure 2. Structures of select BET inhibitors in Table 1 that are not shown in Figure 1 of the main text.
